# Delivery of Native Proteins into *C. elegans* Using a Transduction Protocol Based on Lipid Vesicles

**DOI:** 10.1038/s41598-017-13755-9

**Published:** 2017-11-08

**Authors:** Michele Perni, Francesco A. Aprile, Sam Casford, Benedetta Mannini, Pietro Sormanni, Christopher M. Dobson, Michele Vendruscolo

**Affiliations:** Centre for Misfolding Diseases, Department of Chemistry, University of Cambridge, Cambridge, CB2 1EW UK

## Abstract

The nematode worm *Caenorhabditis elegans* (*C. elegans*) is a versatile and widely used animal model for *in vivo* studies of a broad range of human diseases, in particular for understanding their genetic origins and for screening drug candidates. Nevertheless, the challenges associated with the administration of native proteins to *C. elegans* have limited the range of applications of this animal model in protein-based drug discovery programs. Here, we describe a readily usable protocol for the transduction of native proteins in *C. elegans*, which is based on the encapsulation of the proteins of interest within cationic lipid vesicles, prior to their administration to worms. This procedure limits the degradation of the proteins in the guts of the animals, and promotes their adsorption into body tissues. To illustrate the efficacy of this approach we apply it to deliver an antibody designed to inhibit α-synuclein aggregation, and show that it can lead to the rescue of the disease phenotype in a *C. elegans* model of Parkinson’s disease. As this transduction protocol is fast and inexpensive, we anticipate that it will be readily applicable to protein-based drug discovery studies that utilize *C. elegans* as a model organism.

## Introduction

Transgenic animal models have been playing a major role for several decades in the identification of the mechanisms of onset and progression of many human diseases, in the discovery and validation of drug targets, and in the development of therapeutic compounds^1–4^. In this context, the nematode worm *C. elegans* ^5^ has emerged as a highly effective system for whole-organism high-throughput genetic^6–14^ and drug^15–19^ screening. There are several factors that contribute to the popularity of *C. elegans* for such purposes, including: (1) the cellular complexity and tissue-specific protein expression of the worms are comparable to those of higher organisms (in particular, 38% of worm genes have human orthologues); (2) the worms are small (about 1 mm in length), transparent, and easy to manipulate; (3) they have a short maturation period of about 3 days from egg to adult at 25 °C, and a life span of 2 to 3 weeks; and (4) they are simple and cost-effective to cultivate^5^.

Since *C. elegans* also offers the possibility of detecting the effects of exogenous proteins, such as antibodies and molecular chaperones, as potential drugs, there is a growing interest of the scientific community in the development of high-throughput screening methods of these macromolecules. The applicability of this strategy, however, is currently mostly limited to the generation of transgenic worms^20–22^, which is time consuming, not always successful, and often not suitable in large screening studies.

While a variety of methods of transduction of nucleic acids and proteins are well established for mammalian cells^23,24^, such protocols are not generally applicable to *C. elegans*. Moreover, the thick cuticle of the skin and the presence of proteases and the acidic pH in the gut of the worms make it extremely difficult to administer proteins in this animal model, thus limiting its use for the discovery of potential protein-based therapeutics.

In order to overcome these issues, we describe here a procedure for the administration of macromolecules in *C. elegans* using native proteins as an example. We show that this method allows the effects of protein molecules to be studied *in vivo* in a cost- and time-effective manner. The procedure consists in encapsulating the proteins of interest in lipid vesicles, which are then given to the animals in the nematode growth medium. Similar protocols have been employed successfully for transfecting a variety of mammalian cells, including primary cells, which are notoriously resistant to protein transduction^23,24^.

To illustrate the method we build on recent reports that have shown how *C. elegans* is particularly useful as a model organism for the characterization of the molecular mechanisms underlying ageing and diseases associated with protein misfolding and aggregation^10,17–20^. We demonstrate the efficacy of our method by successfully delivering proteins of different sizes in their native functional states into wild type worms and transgenic worms representing a Parkinson’s disease (PD) model^19,25^. We study four proteins in particular: (1) a readily detectable fluorescent protein (1) mCherry (29 kDa) and (2) a well-established monoclonal antibody YL1/2 (140 kDa) directed against α-tubulin^26–28^, (3) an antibody designed to bind the YFP protein tagged with the Alexa-647 fluorophore, AHP2983^29^, and (4) a single-domain antibody (18 kDa) designed to rationally inhibit the aggregation α-synuclein, a protein linked to the onset and spread of PD and related conditions^30,31^.

## Results and Discussion

### Description of the transduction protocol

**Figure 1:**
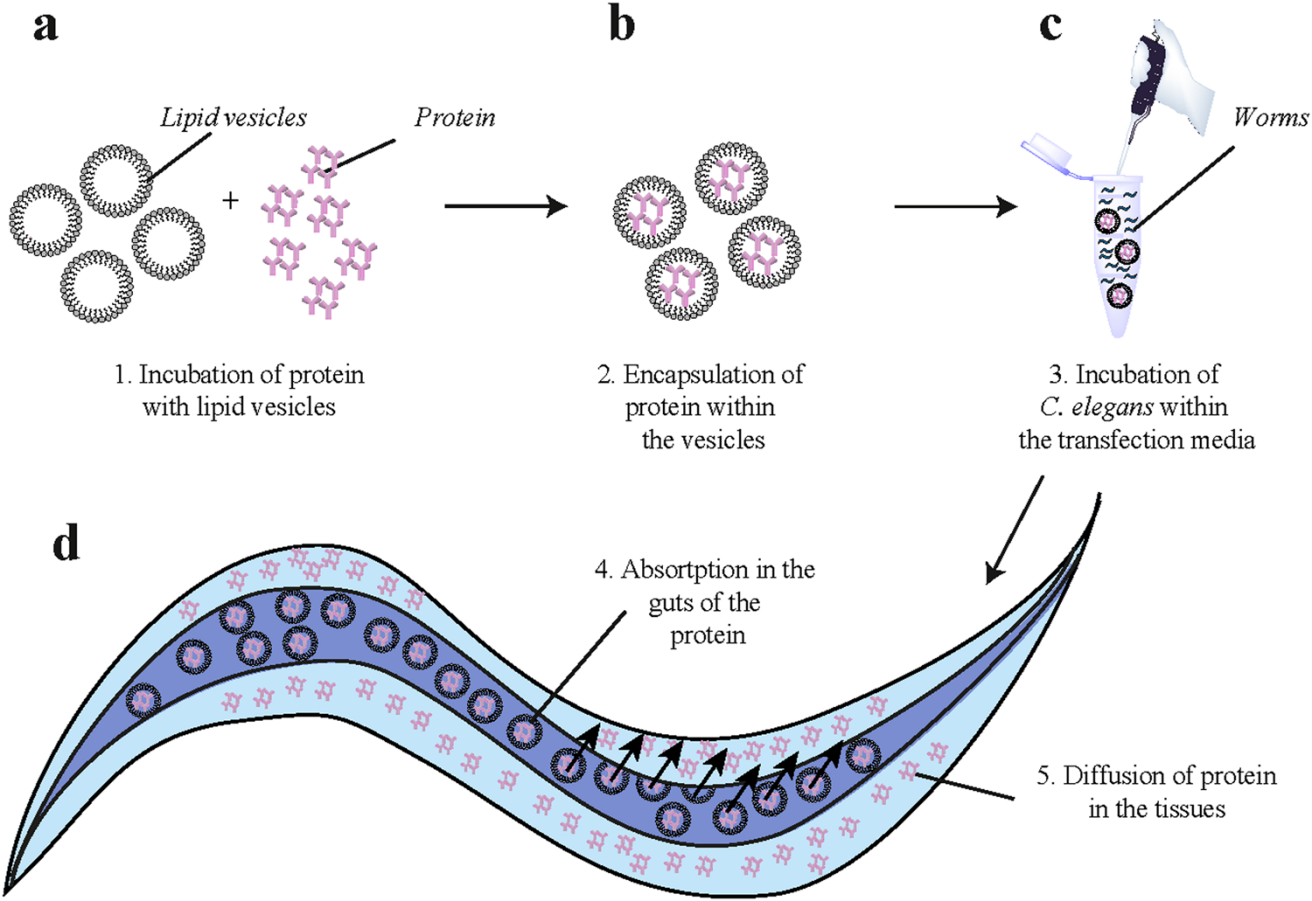
Schematic description of the transduction protocol. (**a**–**d)** Illustration of the four main steps of the protocol: (**a**) Proteins are incubated with the PulsIn lipid mixture transduction medium (see Materials and Methods); (**b**) following incubation, vesicles containing the proteins are formed; (**c**) *C. elegans* are incubated with the vesicles overnight; (**d**) proteins are absorbed in the gut and can diffuse to other tissues of the animals.

The transduction strategy that we developed consists in encapsulating the protein molecules of interest inside cationic lipid vesicles (Fig. 1a,b), with the aim of shielding them from degradation in the acidic environment of the guts of the worms.

The worms are then incubated in a Hepes buffer solution supplemented with these vesicles **(**Fig. 1c). Under these conditions, the animals ingest the vesicles, which are then delivered to the gut; in this location they fuse with the walls of the intestinal lumen and are absorbed into the body tissues, releasing their cargo (Fig. 1d **)**. We coupled this transduction protocol with our recently developed drug discovery screening platform^19^, in order to enable high-throughput antibody screening procedure in *C. elegans*.

### Assessment of the transduction efficiency using a fluorescent marker protein

**Figure 2:**
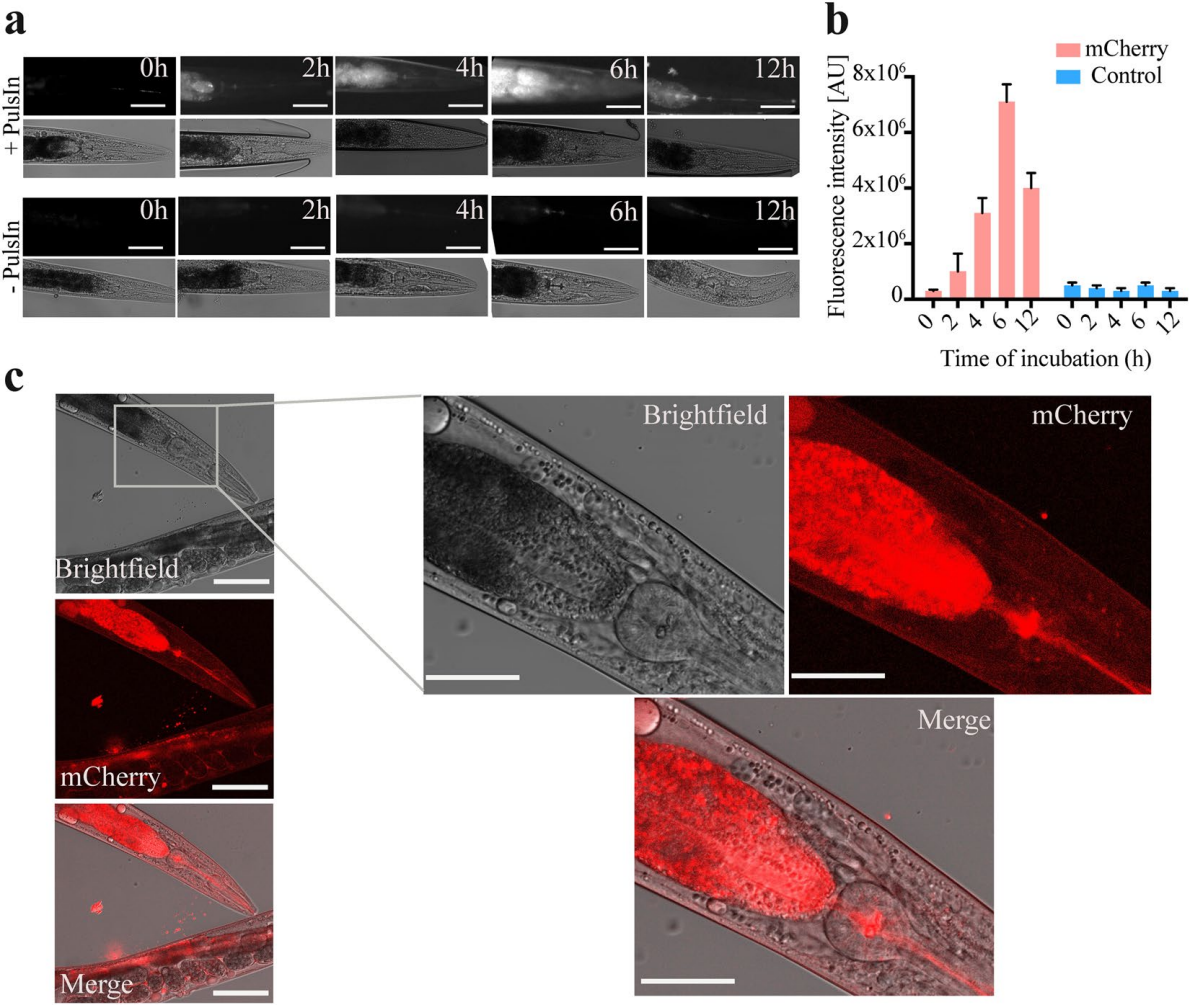
Assessment of the transduction efficiency using a fluorescent protein. (**a**) Fluorescence and bright-field pictures of representative *C. elegans* worms at different incubation times from the treatment (treatment which consisted of a 2–12 hours incubation, see Materials and Methods) with vesicles loaded with the fluorescence marker mCherry (top series) or without vesicles (bottom series). All images were taken with a fluorescence microscopy setup at a magnification of 20X. **(b)** Bar plot showing the mCherry fluorescence signal integrated over the whole worm body at different incubation times from the treatment with the vesicles. (**c)** Confocal microscopy pictures of different part of the body of representative worms were taken at 10X magnification (left panels). Bright-field, fluorescence images and merged image of the two channels are also shown. High magnification images at 20X magnification are also shown (right panels). The error is given as the standard error on the mean (SEM). For quantification, ca. 50 worms were considered. Scale bars represent 80 μm and 40 μm in high magnification images (right panels).

First we established the optimal conditions for protein encapsulation and transduction using the fluorescent proteins mCherry in combination with fluorescence microscopy and confocal microscopy for localisation studies (Fig. 2a–c). mCherry is a 29 kDa fluorescent protein, which absorbs at 587 nm and emits at 610 nm when it is in its native conformation. By using mCherry we could simultaneously track the distribution and the turnover of the protein *in vivo* in the worm bodies (Fig. 2a–c).

Protein solutions at 20 μM concentration were incubated in the presence of 40 μl of a commercial cationic lipid mixture (PulsIn), corresponding to 40–70 μM, for 6 hours (see Materials and Methods). In order to evaluate the efficiency of the encapsulation reaction in these conditions, we also separated the lipid vesicles form the protein/lipid mixture (see **Supplementary Information** and Figure S1) and quantified residual protein fluorescence in the supernatant. We calculated that almost 97% of the protein was encapsulated into lipid vesicles (Figure S1).

For *in vivo* transduction, we used vesicles loaded with mCherry that were then administered to ca. 500 worms in a volume of 1 ml with an incubation time of 6 hours. We chose this treatment time because it was the one at which we could observe the highest fluorescence intensity in the guts of the worms (Fig. 2a,b). All experiments in the present work have been performed using this incubation time, unless otherwise stated. Then, to monitor the protein turnover inside the animals, after 2, 4, 6, 12 hours after transduction, the worms were transferred to 5% agarose pads, and ca. 50 of them (Fig. 2a,b) were analyzed by fluorescence microscopy. After a period of incubation of 2 hours after transduction, mCherry could be seen to be diffusing from the gut to the nearby tissues (Fig. 2a,b). Then, after 4 more hours, mCherry could be observed throughput the whole body of each animal (Fig. 2a,b). Subsequently, after an overall incubation time of 12 hours following transduction, the observed fluorescence was significantly decreased, indicating that degradation of the protein had eventually occurred (Fig. 2a,b). As control, in order to take into account of potential artefacts due to *C. elegans* natural fluorescence, we monitored the fluorescence over time of untreated *C. elegans* worms; in this case, we could not observe any significant signal relative to the background of the plates (Fig. 2a,b). These results confirmed that mCherry was successfully delivered into the guts of worms, following encapsulation into lipid vesicles.

In order to verify the diffusion of mCherry from the guts to the different tissues of the worms, we turned to confocal microscopy (Fig. 2c and S2,3). Confocal microscopy pictures at 6 hours after the treatment of *C. elegans* worms show that the fluorescence of mCherry is observable not only in the guts, but also in all the body mass of the animal (Fig. 2c and S3), proving the diffusion of the protein to different regions of the worm bodies. Finally, in order to determine whether the administration procedure was toxic to the worms, we measured various fitness parameters (namely body bends per minute (BPM), speed, bend amplitude, displacement per bend, and moving fraction) after the treatment. From these measurements we could not observe any significant change in phenotype with respect to untreated worms (Figure S4).

### Application of the administration protocol for localization studies using monoclonal antibodies

We administered to the worms monoclonal antibodies targeting different cellular structures. Our goal was to prove that this administration procedure can be employed to determine the localisation of specific molecules within the worms.

In a first set of experiments, we used a monoclonal antibody anti-α-tubulin, which was labeled with the fluorophore Alexa 647 (see **Material and Methods**). We administered this antibody to the worms and analyzed the Alexa 647 fluorescence after 6 hours from the treatment by means of a light-sheet microscope, which generates 3D reconstructions of fluorescent objects and can readily be used to image *C. elegans* ^32^. We were thus able to obtain 3D images of the worms (a representative one is shown in Figure S5) in which the fluorescence of the anti-α-tubulin antibody was distributed throughout the whole body, as expected. As a control, we carried out the same measurements on worms expressing either a polyglutamine (PolyQ) peptide in fusion with the yellow fluorescent protein YFP (PolyQ:YFP) or expressing the YFP alone in the large muscle cells^33^ (Figure S5). We could indeed observe the localization of aggregated PolyQ:YFP inside the muscle cells, but not observed in other tissues, while YFP alone was observed to be diffused.

**Figure 3:**
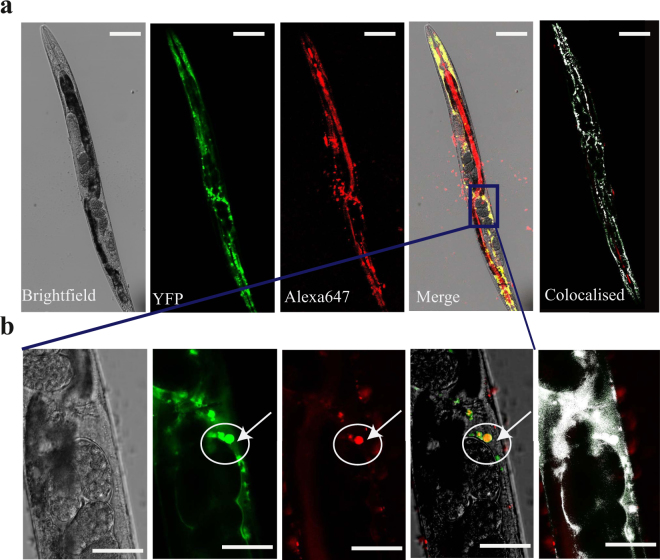
Application of the transduction protocol for localisation studies using monoclonal antibodies. (**a**) Confocal microscopy pictures of a representative poly-Q:YFP *C. elegans* worm treated with vesicles loaded with an Alexa647-monoclonal antibody targeting the YFP. Different channels are shown: Bright-field, YFP fluorescence, Alexa647, merge of the fluorescence channels and overlay of all channels are shown. (**b**) Close-up image of the aggregates in panel b acquired at 20X magnification; same channels are shown. For colocalization images at low magnification (top panel) r = 0.21 and tM1 = 0.38 and tM2 = 0.54 in the low magnification images (top row)'. For images at high magnification r = 0.55 and tM1 = 0.37 and tM2 = 0.64 (bottom row). Scale bars represent 80 μm and 40 μm in the high magnification images.

As a further proof of the efficacy of our method, in addition to the localization of α-tubulin, we investigated by confocal microscopy also the localization of aggregates made of the polyQ:YFP fusion protein. We then applied the same administration procedure using an antibody against YFP tagged with Alexa 647 (Fig. 3a). In this case, we found that the fluorescence pattern of the anti-YFP antibody was extremely different from the one observed in the previous experiment. In particular, the fluorescence of the antibody was not diffused but clustered within the body of the animals and showed a very good co-localisation (Fig. 3a,b) with the signal of the PolyQ:YFP protein (Pearson’s coefficient of correlation r = 0.21 and Manders’ Coefficients tM1 = 0.38 and tM2 = 0.54); to quantify the colocalization of the two signals, we used the imaging analysis tool ImageJ “Colocalisation threshold”^34^. This last result in particular provides further evidence regarding the fact that a protein administered with our method is able to successfully diffuse from the guts to the different tissues of the animal in its native and functional state.

### Application of the transduction protocol for drug discovery using an antibody designed against α-synuclein

**Figure 4:**
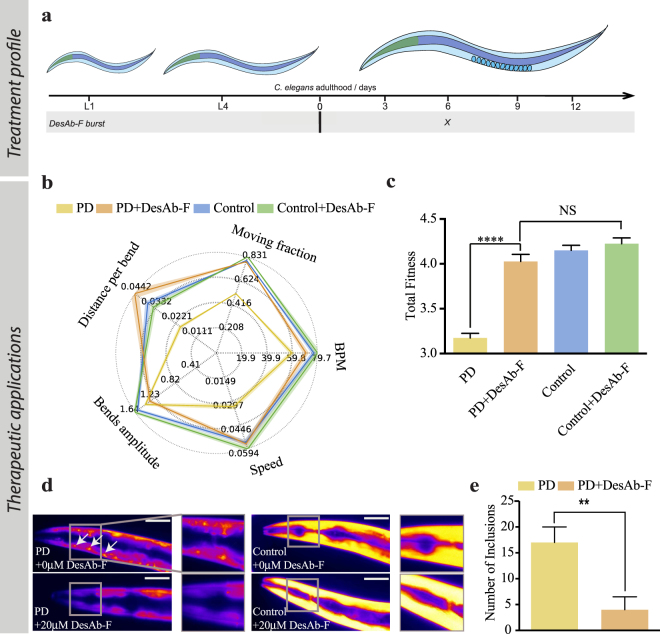
Application of the transduction protocol for drug discovery using an antibody against α-synuclein. (**a**) PD worms at day 6 of adulthood were incubated for 6 hours with 20 μM DesAb-F in hepes buffer. Their motility was then assessed at day 7 of adulthood. (**b)** Fingerprint map and (**c**) total fitness analysis showing the effects of DesAb-F on the worm fitness. Administration of 20 μM DesAb-F led to a significant recovery of worm fitness (p < 0.005). (**d**) Left, representative images of PD worms with and without 20 μM of DesAb-F, respectively; right side shows that the localization of YFP in control worms was not affected by the addition of DesAb-F. Inserts show zoomed head regions that highlight the α-synuclein inclusions. The YFP signal was false coloured for the fluorescence intensity. (**e**) Quantification of the number of inclusions with or without the addition of 20 μM of DesAb-F, respectively. Error represents Standard error on the mean (SEM). BPM indicates Body Bend per Minute (BPM). Scale bars represent 80 μm. The double (**) and quadruple asterisk (****) indicates p < 0.05 and p < 0.0005, respectively (Student’s t test).

In order to test the transduction protocol, we used it to administer a rationally designed anti-aggregation single-domain antibody, called DesAb-F^35^, to a PD worm model (Fig. 4). In this model α-synuclein:YFP is over-expressed in the large muscle cells, where it forms inclusions and leads to age-dependent motility dysfunction^19,25^. We administered DesAb-F at day 6 of adulthood of the PD animals, when the toxicity of α-synuclein aggregates can clearly be observed^19^, and we monitored the effect of the antibody after 12 h from the treatment (Fig. 4), because at this time untreated and treated worms exhibited a large difference in fitness (Figure S6). DesAb-F induced a significant recovery of fitness (Fig. 4b,c), and the effect was clear in several different worm phenotypes, such as thrashing, speed of movement, paralysis rate, bends amplitude and distance per bend, analyzed with our automated platform^19^ (Fig. 4b). The mobility of the control animals expressing only YFP, treated with empty vesicles, was not significantly affected with treatment by DesAb-F (Fig. 4b), thus indicating a highly specific effect of this antibody towards α-synuclein aggregation. To validate further possible secondary effects of the treatment, we tested the effects of the administration of empty vesicles on the fitness of wild-type and α-synuclein worms. We thus observed a slight improvement of the physiological parameters of the worms, which, however, was significantly lower than that observed upon treatment with vesicles loaded with DesAb-F (Figure S7).

To further validate these therapeutic effects observed *in vivo*, we also visualized the α-synuclein:YFP inclusions via fluorescent microscopy and observed a significantly decreased amounts of α-synuclein inclusions upon treatment with the mixture containing both the lipids and the peptide (Fig. 4d,e). We also note that the expression patterns of control YFP worms treated with empty vesicles were not affected by the DesAb-F administration.

## Conclusions

We have reported the development and application of a protocol to deliver native proteins, including antibodies, in *C. elegans* worms. Despite the widespread use of these worms in the biological and medical fields, effective systems for the delivery of active biological macromolecules into them are still not generally available. Therefore, it is currently typically necessary to create transgenic animals to deliver proteins *in vivo*.

Our approach, which was prompted by procedures already well established for mammalian cells, can effectively deliver proteins to the internal tissues of the worms by encapsulating them into lipid vesicles, which are then added directly to the growth medium. We directly visualized and measured the efficacy of this transduction protocol by using the fluorescent market mCherry, an anti-α-tubulin antibody, and an anti-YFP antibody. We then tested the therapeutic possibilities of this approach by showing a significant recovery of the pathological phenotype in a PD worm model following delivery of an antibody designed to inhibit α-synuclein aggregation.

We anticipate that this protocol will provide a simple, cost effective and rapid method for the delivery of native proteins that could be used as an alternative to the creation of transgenic worms. This method could therefore serve to extend the scope to use this model organism for a wider range of applications, including protein design and antibody discovery.

## Materials and Methods

### Media

Standard conditions were used for the propagation of *C. elegans* ^19^. Briefly, the animals were synchronized by hypochlorite bleaching, hatched overnight in M9 (3 g/l KH2PO4, 6 g/l Na2HPO4, 5 g/l NaCl, 1 µM MgSO4) buffer, and subsequently cultured at 20 °C on nematode growth medium (NGM) (CaCl2 1mM, MgSO4 1 mM, cholesterol 5 µg/ml, 250 µM KH2PO4 pH 6, Agar 17 g/l, NaCl 3g/l, casein 7.5g/l) plates seeded with the *E. coli* strain OP50. Saturated cultures of OP50 were grown by inoculating 50 mL of LB medium (tryptone 10 g/l, NaCl 10 g/l, yeast extract 5 g/l) with OP50 and incubating the culture for 16 h at 37 °C. NGM plates were seeded with bacteria by adding 350 µl of saturated OP50 to each plate and leaving the plates at 20 °C for 2–3 days. On day 3 after synchronization, the animals were placed on NGM plates containing 5-fluoro-2′deoxy-uridine (FUDR) (6.83 nM, unless stated otherwise) to inhibit the growth of offspring. FUDR plates were seeded with bacteria by adding 350 µl of 10x concentrated OP50 solution to ensure starvation did not occur for the lifespan of the worm. Concentrated OP50 solution was obtained by centrifuging 1 L of saturated OP50 culture at 5000 RPM for 15 minutes and suspending the resultant pellet in 100 mL sterile water.

### Tracking analysis

Analysis was carried out as described previously^19^. Briefly, we used custom software written in Python (Python Software Foundation) called the WF-NTP (Wide Field-of-view Nematode Tracking Platform)^19^. Our code initially detects and subtracts the background, consisting of non-moving objects such as small particles and shadows from the agar plate. After this operation, the remaining labeled regions are identified as individual worms and the positions of such regions are then stored for each frame. The eccentricity of each tracked worm, a measure of the ratio of the major and minor ellipse axes, can then be used to estimate worm body bending as a function of time. Through this method, individual worms can be tracked over time, and plots of their movement can be extracted to give visual information about their mobility levels.

### Strain

The following strains were used: zgIs15 [P(unc-54)::αsyn::YFP]IV (OW40). In OW40, α-synuclein fused to YFP relocates to inclusions, which are visible as early as day 2 after hatching and increase in number and size during the aging of the animals, up to late adulthood (Day 17)^19,25^; rmIs126 [P(unc-54)Q0::YFP]V (OW450). In OW450, YFP alone is expressed and remains diffusely localized throughout ageing^25^. The strains were kindly provided by Prof. Ellen Nollen, European Research Institute of the Biology of Ageing (ERIBA) (Groningen, The Netherlands); C. *elegans* var Bristol (N2) used as wild type strain^5^.

### Automated motility assay on agar plates

All *C. elegans* populations were cultured at 20 °C and developmentally synchronized by hypochlorite bleaching. Unsynchronized animals were washed off NGM plates using M9 buffer and centrifuged at 2000 RPM for 2 min, the supernatant was removed to leave 2 mL of M9. 1 mL of hypochlorite bleaching solution was then added and the mixture agitated for 210 s before being diluted to 15 mL using M9, animals were then subjected to 5 rounds of centrifugation at 2000 RPM for 2 min followed by removing the supernatant and suspending in 15 mL of M9 to dilute out the hypochlorite bleaching solution. After 5 washing cycles the animals were transferred to 12 well tissue culture plates and allowed to hatch overnight, development was arrested at L1 larval stage due to a lack of food. The now synchronized animals were then transferred to OP50 seeded NGM plates and allowed to develop for 64–72 hours before being transferred to FUDR plates. At defined ages, the animals were washed off the plates with M9 buffer and incubated with specific antibodies for 6 hours. Worms were then transferred on FUDR plates and let recover overnight. The morning after, the worms were spread over an OP-50 un-seeded 9 cm plate, after which their movements were recorded at 20 fps using a rationally designed microscopic method, for 2min^19^. Up to 1500 animals were screened per condition per time point for each experiment unless stated otherwise. One experiment that is representative of the three measured is shown in the figure. Videos were analysed using a custom made tracking code^19^.

### Transduction protocol

About 500 *C. elegans* worms were incubated in M9 with 20 μM mCherry (ABE3463) (Bioscience Lifesciences, Cambridge, UK), an α-tubulin antibody (YL1/2) [Alexa Fluor (R) 647] (Novus Biologicals, Littleton, CO, USA)^26–28^, and antibody against YFP [AHP2983, (Bio-Rad, Hercules, California, USA)^29^, and DesAb-F^35^, and 40 μl PulsIn (40–70 μM) (PolyPlus tranfection SA, Illkirch-Graffenstaden, France) in a final volume of 1 mL. To tag Alexa 647 to the AHP2983 an Alexa 647 labelin kit was used [A20186] (Invitrogen, Carlsbad, California, USA). Imaging procedures were carried out 2 to 12 h after transduction. Motility procedures were carried between 6 to 84 hours after transduction. All experiments were carried out in triplicate. One experiment that is representative of the three independent ones measured is shown in the figure. As control we considered worms treated with empty vesicles, to take into consideration possible effects of the lipids on worm behavior.

### Quantification of inclusions

To monitor the number of inclusions in each worm, individual animals were mounted on 5% agarose pads and anaesthetized using 1% NaN_3_, on glass microscope slides for imaging. For quantification of the number of inclusions in α-synuclein:YFP animals, only the frontal region of the worms was considered^25^. The number of inclusions in each animal was counted using a Leica MZ16 FA fluorescence dissection stereomicroscope (Leica Microsystems, Wetzlar, Germany) at a nominal magnification of 20X or 40X^19^. At least 50 animals were examined per condition, unless stated otherwise. All experiments were carried out in triplicate and the data from one representative experiment are shown in the figure. The Student’s t-test was used to calculate p-values, and all tests were two-tailed unpaired unless otherwise stated.

### Imaging

The fluorescence of mCherry in the worm bodies was quantified using a Leica MZ16 FA fluorescence dissection stereomicroscope (Leica Microsystems, Wetzlar, Germany) at a nominal magnification of 20X or 40X, and images were acquired using an Evolve512 Delta EMCCD Camera, with high quantum efficiency (Photometrics, Tucson, AZ, USA). Measurements and quantifications were performed using ImageJ software (National Institutes of Health, Bethesda, MD, USA). Light-sheet imaging was carried out using a custom-built Light Sheet Fluorescence Microscope (Cambridge University Imaging Centre, CUIC, Cambridge, UK) and using an omicron laser combiner with excitation at 488 nm for YFP and 638 nm for Alexa 647. Detection was carried out using a Hamamatsu Orca Flash 4 camera. 200 planes of 1 μm each were acquired per image. For confocal microscopy a Leica SP8 (Leica Microsystems, Wetzlar, Germany) was used at a nominal magnification of 10 or 20X. ImageJ software (National Institutes of Health, Bethesda, MD, USA) plug-In colocalization threshold was used for colocalization quantifications^34^.

## Electronic supplementary material

Supplementary Information
