# Supplementary material for: Delivery of Native Proteins into C. elegans Using a Transduction Protocol Based on Lipid Vesicles
Source: Sci Rep. 2017 Nov 8;7:15045. doi: 10.1038/s41598-017-13755-9 (PMC5678089; doi:10.1038/s41598-017-13755-9)
Supplement: Supplementary file 1 — Supplementary Information [file 41598_2017_13755_MOESM1_ESM.pdf]

Supplementary Material for:
Delivery of Native Proteins into *C. elegans* Using a Transduction Protocol Based on Lipid Vesicles

Michele Perni, Francesco A. Aprile*, Sam Casford, Benedetta Mannini,
Pietro Sormanni, Christopher M. Dobson and Michele Vendruscolo*

Centre for Misfolding Diseases, Department of Chemistry,
University of Cambridge, Cambridge CB2 1EW, UK

Extended Experimental Methods

Vesicle purification via sucrose gradient: For measurement of fluorescence efficiency and PulsIn encapsulation, we adapted the protocol recently described by Cox and Tompson[36]. Briefly a sucrose gradient consisting of 240 ul of 1M sucrose, 240 μl 0.8 Sucrose, 240 μl 0.6 sucrose and 180 μl 0.4M sucrose was formed and 60ul of samples, which consisted in 40 μl PulsIn and 20ul hepes, or Phycoeritrin or PulsIn Only were incubated. Samples were then ultracentrifuged at 100.000g using a Beckman counter Optima TLX 120.2 for 2 h. Resulting phases, either the protein fraction (below 1M), consisting in the non-encapsulated protein or the lipids fraction (0.6-0.8M), consisting of the protein encapsulated in vesicles, were separated and flowingly analysed with a Cary Eclipse Fluorimeter by acquiring spectra with an excitation at 488nm. For quantification the main peak at 575nm was considered.

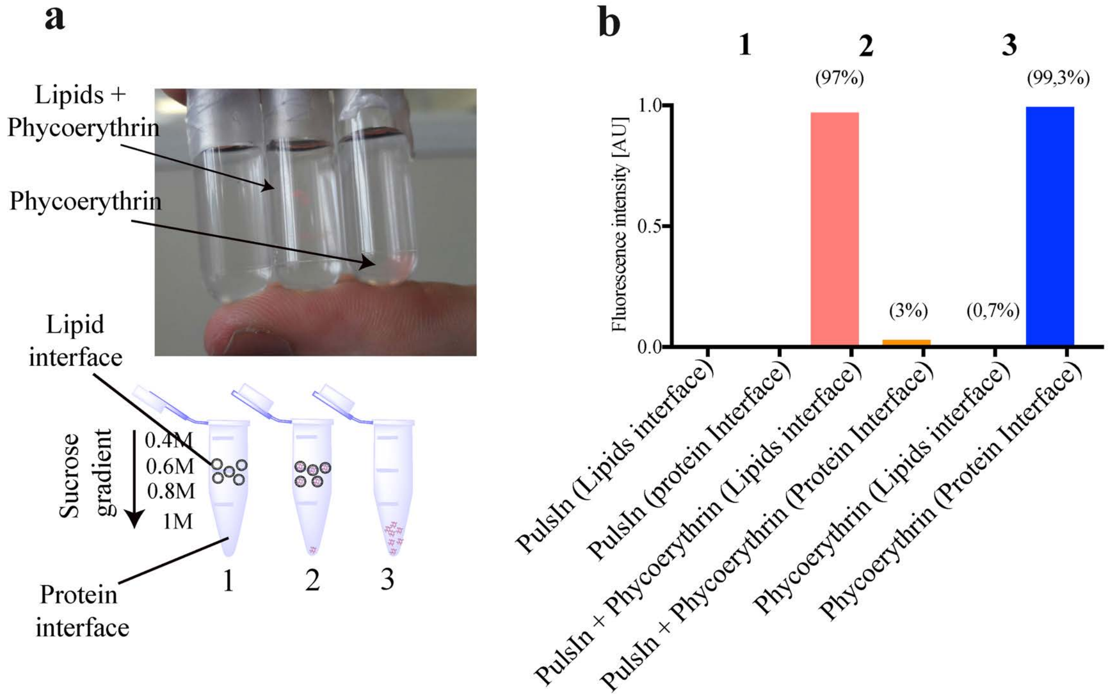

Figure S1. Efficiency of encapsulation of PulsIn vesicles using the protocol discussed in this work. (**a**) Picture and schematic representation of the experimental setup. (**b**) Bar plot showing the fluorescence of the protein in the supernatant after sucrose - separation of the liposome fraction from the mixture. Fluorescence intensities of pellets and lipids containing interphases of samples containing 1) vesicles only (grey), 2) phycoerythrin and and PulsIn (red) and 3) phycoerythrin only (blue) incubated for 6 hours have been normalized over the total fluorescence of each individual sample. Relative rations corresponding to the different fractions are shown.

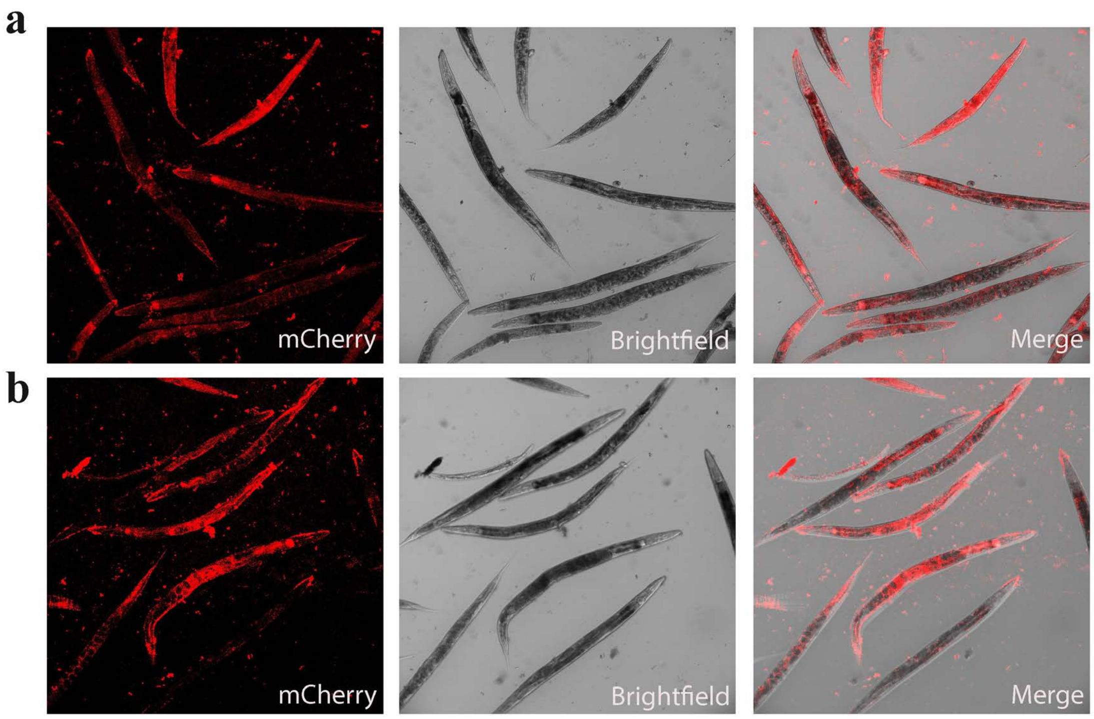

Figure S2: Representative images of worms transducted with mCherry. (a-b) Fluorescence microscopy showing multiple worms transducted with 20 μM mCherry and PulsIn for 6 hours. The mCherry, brightfield and merged channels are shown (Left, centre, and right, respectively).

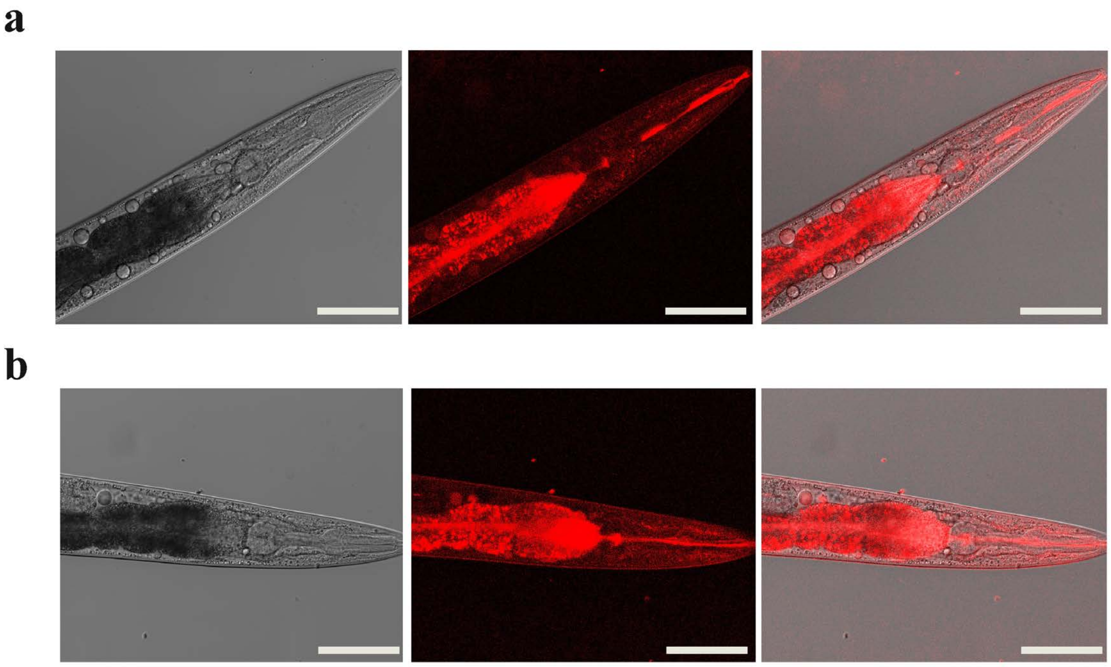

Figure S3: Confocal pictures of worms transducted with mCherry. Confocal microscopy showing worms transducted with 20 μM mCherry and PulsIn for 6 hours. Scale bars represent 80 μm.

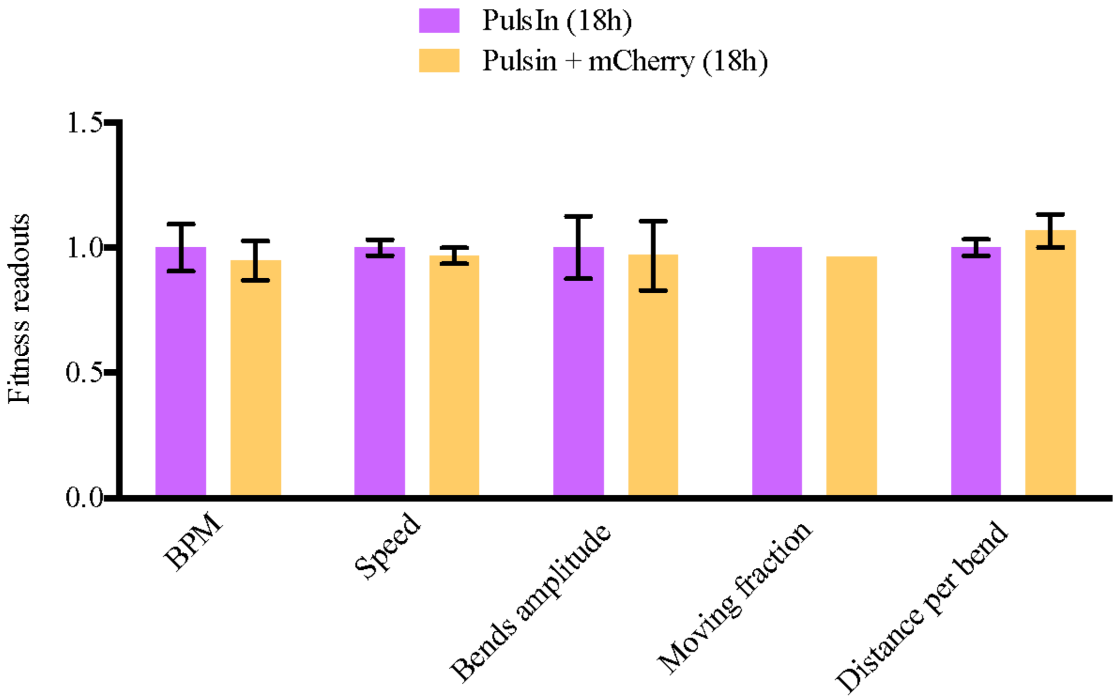

Figure S4. Effects of the transduction of mCherry on the viability of the worms. Bar plot showing multiple fitness readouts of worms treated with or without mCherry (body bends per minute (BPM), Speed, Bends amplitude, Displacement per bend, Moving Fraction). Ca. 500 worms were transduced for 6 hours with 20μM mCherry. After this, the worms were transferred on FUDR plates and let equilibrate overnight (for ca. 12 hours) prior to the phenotype screening. The error is given as the standard error on the mean (SEM).

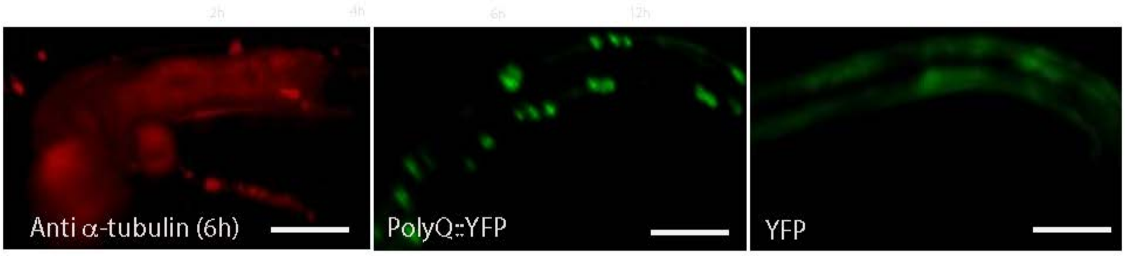

Figure S5. Application of the transduction protocol for localisation studies using monoclonal antibodies. Light sheet microscopy picture of wild type *C. elegans* worms treated with vesicles loaded with a monoclonal antibody targeting α-tubulin (left panel), YL1/2. The YL1/2 is conjugated to Alexa 647 fluorophore, which gives the fluorescence signal. Poly-Q: YFP worms (central panel) and YFP worms (right panel) are shown as control.

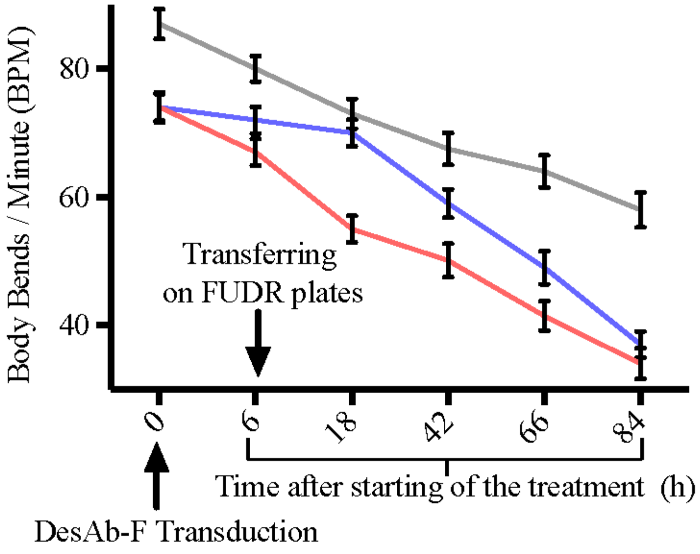

Figure S6. Screening of incubation times after treatment. Body bends per minute (BPM) of *C. elegans* worms expressing α-synuclein (PD, red), treated with vesicles loaded with the antibody DesAb-F (blue) and control wild type worms treated with empty vesicles (grey). Error bars represent the standard error of the mean (SEM).

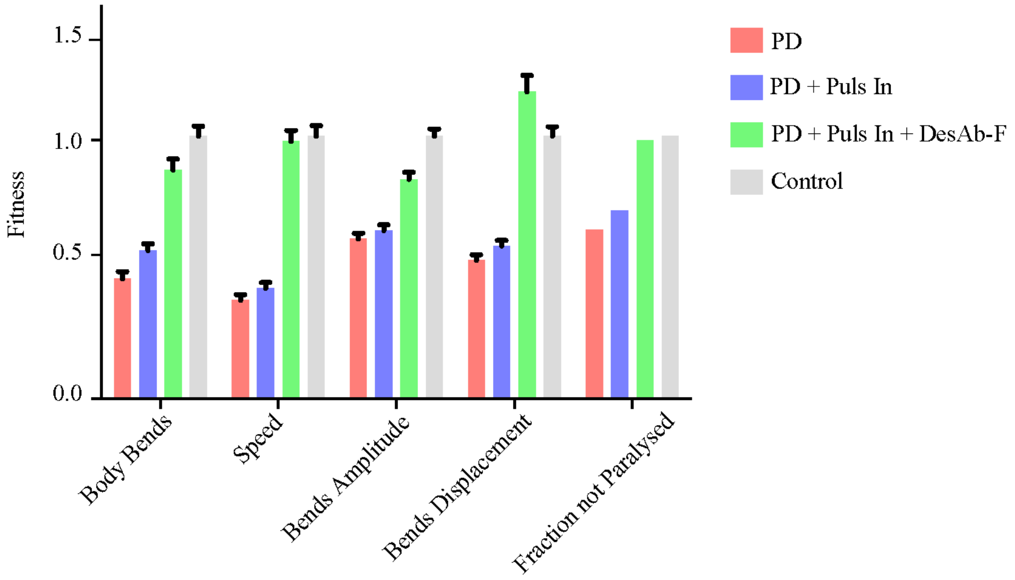

Figura S7. Effect of empty vesicles (PulsIn) on different fitness parameters of the worms. Different fitness parameters of untreated PD worms (red), treated with empty vesicles (blue), treated with vesicles loaded with the antibody DesAb-F (green). Same fitness parameters of wild type worms (grey) are shown as a control. Error bars represent the standard error on the mean (SEM).
